# Invasive vs. conservative management of older patients with non-ST-elevation acute coronary syndrome: individual patient data meta-analysis

**DOI:** 10.1093/eurheartj/ehae151

**Published:** 2024-04-10

**Authors:** Christos P Kotanidis, Gregory B Mills, Bjørn Bendz, Erlend S Berg, David Hildick-Smith, Geir Hirlekar, Dejan Milasinovic, Nuccia Morici, Aung Myat, Nicolai Tegn, Juan Sanchis, Stefano Savonitto, Stefano De Servi, Keith A A Fox, Stuart Pocock, Vijay Kunadian

**Affiliations:** Translational and Clinical Research Institute, Faculty of Medical Sciences, Newcastle University, 4th Floor William Leech Building, Newcastle upon Tyne NE2 4HH, UK; Cardiothoracic Centre, Freeman Hospital, Newcastle upon Tyne Hospitals NHS Foundation Trust, Newcastle upon Tyne, High Heaton NE7 7DN, United Kingdom; Translational and Clinical Research Institute, Faculty of Medical Sciences, Newcastle University, 4th Floor William Leech Building, Newcastle upon Tyne NE2 4HH, UK; Cardiothoracic Centre, Freeman Hospital, Newcastle upon Tyne Hospitals NHS Foundation Trust, Newcastle upon Tyne, High Heaton NE7 7DN, United Kingdom; Department of Cardiology, Oslo University Hospital, Oslo, Norway; Institute of Clinical Medicine, University of Oslo, Oslo, Norway; Department of Cardiology, Oslo University Hospital, Oslo, Norway; Institute of Clinical Medicine, University of Oslo, Oslo, Norway; Sussex Cardiac Centre, University Hospitals Sussex NHS Foundation Trust, Brighton, UK; Department of Molecular and Clinical Medicine, Institute of Medicine, Gothenburg University, Gothenburg, Sweden; Department of Cardiology, Sahlgrenska University Hospital, Gothenburg, Sweden; Department of Cardiology, University Clinical Center of Serbia, Belgrade, Serbia; Medical Faculty, University of Belgrade, Belgrade, Serbia; IRCCS Fondazione Don Carlo Gnocchi, Milan, Italy; Medpace UK, London, UK; Department of Cardiology, Oslo University Hospital, Oslo, Norway; Institute of Clinical Medicine, University of Oslo, Oslo, Norway; Department of Cardiology, Hospital Clinico Universitario, INCLIVA, Universitat de Valencia, CIBER-Cardiovascular, Valencia, Spain; Clinica San Martino, Malgrate, Italy; Department of Molecular Medicine, University of Pavia, Pavia, Italy; Centre for Cardiovascular Science, University of Edinburgh, Edinburgh, UK; London School of Hygiene and Tropical Medicine, London, UK; Translational and Clinical Research Institute, Faculty of Medical Sciences, Newcastle University, 4th Floor William Leech Building, Newcastle upon Tyne NE2 4HH, UK; Cardiothoracic Centre, Freeman Hospital, Newcastle upon Tyne Hospitals NHS Foundation Trust, Newcastle upon Tyne, High Heaton NE7 7DN, United Kingdom

**Keywords:** Acute coronary syndrome, Coronary angiography, Myocardial infarction, Older adults, Percutaneous coronary intervention

## Abstract

**Background and Aims:**

Older patients with non-ST-elevation acute coronary syndrome (NSTEACS) are less likely to receive guideline-recommended care including coronary angiography and revascularization. Evidence-based recommendations regarding interventional management strategies in this patient cohort are scarce. This meta-analysis aimed to assess the impact of routine invasive vs. conservative management of NSTEACS by using individual patient data (IPD) from all available randomized controlled trials (RCTs) including older patients.

**Methods:**

MEDLINE, Web of Science and Scopus were searched between 1 January 2010 and 11 September 2023. RCTs investigating routine invasive and conservative strategies in persons >70 years old with NSTEACS were included. Observational studies or trials involving populations outside the target range were excluded. The primary endpoint was a composite of all-cause mortality and myocardial infarction (MI) at 1 year. One-stage IPD meta-analyses were adopted by use of random-effects and fixed-effect Cox models. This meta-analysis is registered with PROSPERO (CRD42023379819).

**Results:**

Six eligible studies were identified including 1479 participants. The primary endpoint occurred in 181 of 736 (24.5%) participants in the invasive management group compared with 215 of 743 (28.9%) participants in the conservative management group with a hazard ratio (HR) from random-effects model of 0.87 (95% CI 0.63–1.22; *P* = .43). The hazard for MI at 1 year was significantly lower in the invasive group compared with the conservative group (HR from random-effects model 0.62, 95% CI 0.44–0.87; *P* = .006). Similar results were seen for urgent revascularization (HR from random-effects model 0.41, 95% CI 0.18–0.95; *P* = .037). There was no significant difference in mortality.

**Conclusions:**

No evidence was found that routine invasive treatment for NSTEACS in older patients reduces the risk of a composite of all-cause mortality and MI within 1 year compared with conservative management. However, there is convincing evidence that invasive treatment significantly lowers the risk of repeat MI or urgent revascularisation. Further evidence is needed from ongoing larger clinical trials.

## Structured Graphical Abstract

**Structured Graphical Abstract:**
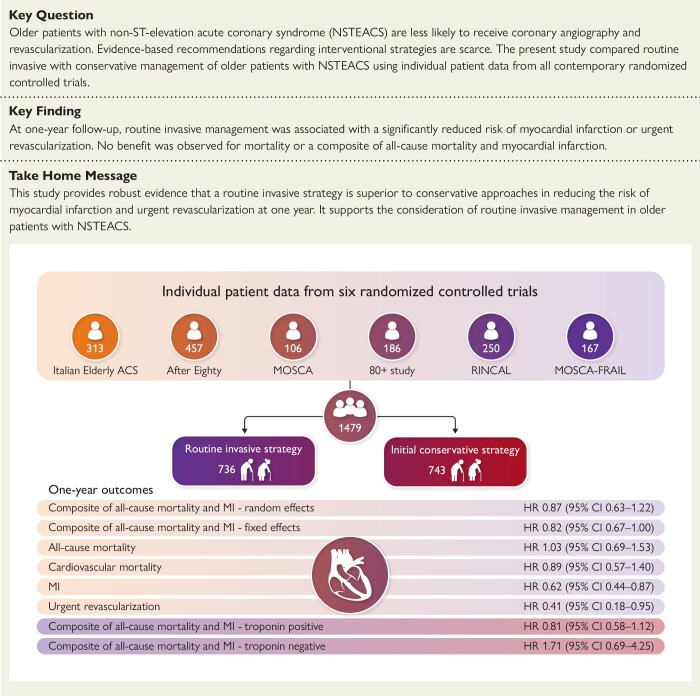
Using individual patient-level data we show that the risk of myocardial infarction and unplanned urgent revascularization is lower in older patients with non-ST-elevation acute coronary syndrome (NSTEACS) treated with a routine invasive strategy compared to a conservative medical approach. The risk of a composite endpoint of all-cause mortality and reinfarction showed weaker evidence of a potentially lower risk for an invasive strategy, while we observed no evident difference for all-cause mortality, cardiovascular death, and stroke. CI, confidence interval; HR, hazard ratio; MI, myocardial infarction.

**See the editorial comment for this article ‘An initial invasive strategy in older patients with non-ST elevation acute coronary syndromes: it is never too late', by J. J. Coughlan *et al*., https://doi.org/10.1093/eurheartj/ehae255.**

## Introduction

The global population aged over 65 years is forecast to double to 1.5 billion people by 2050.^1^ Ischaemic heart disease is the leading cause of death worldwide, with the greatest mortality burden afflicting older adults.^2^ International treatment guidelines recommend coronary angiography with subsequent revascularization if indicated in higher-risk patients following non-ST-elevation acute coronary syndrome (NSTEACS),^3^ yet evidence-based recommendations for the care of older patients with NSTEACS are scarce—this is highlighted as a significant gap in evidence.^4–6^ This patient group is at greatest baseline risk of in-hospital death, ischaemic events, and bleeding, yet paradoxically is under-represented in clinical trials of acute coronary syndrome treatment strategy and least likely to receive evidence-based medications, coronary angiography, and revascularization.^7–10^

Although limited by selection bias, registry data, and subgroup analyses suggest older patients with NSTEACS are likely to gain a net clinical benefit from an invasive approach.^11^ Current guidelines are based on historical landmark studies, such as the FIR trials,^12^ that may not reflect the heterogeneous older, ageing, and significantly co-morbid population encountered in clinical practice today. Subgroup analyses have not been adequately powered and reflect standards of care before radial access, drug-eluting stents and modern dual antiplatelet therapy strategies demonstrated superior outcomes. However, only six randomized controlled trials (RCTs) have been published comparing an interventional vs. conservative approach in older patients with NSTEACS. They have been underpowered and produced inconsistent findings with limited generalizability due to absence of frailty and co-morbidity assessments.^13–18^ The largest trial, the After Eighty study, found significantly lower rates of reinfarction and urgent revascularization at 18 months but no mortality benefit.^14^

Given the paucity of evidence, there is considerable discrepancy in how older NSTEACS patients are managed which could ultimately lead to a sizeable proportion of this patient subpopulation being withheld prognostically beneficial interventional treatment. The aim of this meta-analysis is to investigate the benefit of a routine invasive strategy in comparison to a conservative strategy among older adults using individual patient data (IPD) from all recent RCTs.

## Methods

### Search strategy and selection criteria

Studies were identified by searching MEDLINE, Web of Science and Scopus (from database inception up to 11 September 2023) with no language restrictions. The detailed search algorithm is given in the Supplementary data online, *Appendix* (p3). In addition, researchers were contacted for any other data sources, including unpublished studies. We also hand-searched trial registries and reference lists of included trials for completeness. Once duplicates were removed, studies were assessed for eligibility by two reviewers (C.P.K. and G.B.M.). Eligible studies were RCTs specifically investigating a routine invasive strategy compared with an optimal medical or conservative approach in older patients with NSTEACS. Any conflicts were resolved through discussion between the reviewers. Lead authors of eligible articles were contacted via e-mail with a request to share published and/or unpublished IPD from their trial. We received a response from all six eligible studies included in the IPD meta-analysis. This IPD meta-analysis was registered on PROSPERO (CRD42023379819) and is reported according to PRISMA-IPD guidance.

### Study eligibility, comparison groups, and outcomes

Eligible participants were patients admitted with NSTEACS aged over 70 years. NSTEACS was defined, according to international guidelines in place at the time of each trial, as ischaemic symptoms (i.e. chest pain) within the previous 72 h with ischaemic ST-segment depression and/or elevated troponin I, troponin T, or creatine kinase-myocardial band.

Participants were randomized in all included trials as receiving ‘early invasive’ or ‘early conservative’ management. Early invasive management comprised coronary angiography within 72 h and, when indicated, coronary revascularization by either percutaneous coronary intervention (PCI) or coronary artery bypass graft (CABG) according to coronary anatomy, patient preference, and institutional algorithms, as well as guideline-directed medical therapy. Conservative management (reference group) consisted of guideline-directed medical therapy only, with coronary angiography during index hospitalization reserved only for clinical indications as permitted by each individual trial, including refractory ischaemia, recurrent myocardial infarction (MI), heart failure of ischaemic origin, or malignant ventricular arrhythmias.

The primary outcome was a composite of all-cause mortality and MI at 1 year. All studies defined MI as new cardiac symptoms with troponin > 99th percentile (specific definitions shown in Online Supplementary data online, *Table S1* and *Appendix* p6). Four studies included peri-procedural myocardial infarctions (MIs). Secondary outcomes included all-cause mortality, cardiovascular death, MI, urgent revascularization, and stroke as originally defined by the included studies (see Supplementary data online, *Appendix* p6). Follow-up time was censored at 1 year post-recruitment for all outcomes across all studies.

Baseline characteristics including age, sex, smoking, angina, previous MI, previous CABG, previous PCI, hypertension, diabetes mellitus, previous stroke, peripheral arterial disease, atrial fibrillation, Killip class, height, weight, medications at discharge, and time to event outcomes, including all-cause mortality, cardiovascular mortality, MI, stroke, and repeat revascularization were extracted at the individual level. Data accuracy was ensured by replicating all published results for all included trials and verified by two independent reviewers (CPK and GBM).

### Risk of bias assessment

The Cochrane Collaboration’s tool (RoB 2) was used for assessing risk of bias.^19^ This assesses bias across multiple domains including bias arising from the randomization process, bias due to deviations from intended interventions, bias due to missing outcome data, bias in measurement of the outcome, bias in selection of the reported result.

### Statistical analysis

The provided data were assessed for completeness and integrity before being combined into one database. The primary analyses compared the risk of adverse outcomes in patients receiving early invasive management with that in patients initially managed medically by using unadjusted (HR) and adjusted (aHR) hazard ratios with 95% confidence intervals (CI). A one-stage, random-effects, IPD meta-analysis was done for each outcome based on the treatment group by use of Cox models with random-effects associated with both the study and the treatment, capturing variability in the treatment effect across studies. A one-level stratified Cox regression model was adopted for fixed effect HRs. No further variables were adjusted for in our main analysis as all trials were RCTs. The time to outcome was based on the time of enrolment and censored for all participants at 365 days. We undertook an intention-to-treat approach to our analysis. The proportional hazards assumption test was performed before all analyses were undertaken to ensure that hazards associated with the treatment effect remain proportional over time.

For all main analyses, only variables with missingness <10% were considered. For continuous variables, missing values were replaced by the mean value, whereas for categorical variables we created an extra level to depict a missing value. Sensitivity analyses in the appendix include models adjusted for age, sex, hypertension, and diabetes. Subgroup analyses were planned *a priori* for age groups, sex, diabetes mellitus status, previous PCI, and post-hoc for previous CABG and troponin positivity. Interaction *P*-values were obtained by adding an interaction term of the treatment category and the factor variable tested in the Cox model. We also present scatterplots of the effect size for the primary endpoint with study-level crossover rates of revascularization during the index hospitalization and publication year to visually explore potential sources of heterogeneity and assess impact on the overall treatment effect estimates. Finally, for completeness of our analytical approach, we conducted a two-stage approach, by calculating individual HRs for each trial in the first stage, and applying a DerSimonian-Laird random-effects synthesis approach to combine the effect sizes obtained from individual studies in the second stage using the ‘rma’ function from the metafor package. Analyses were performed in the R environment (R version 3.6.0, R Studio version 1.2.1335 with ‘survival’, ‘coxme’, ‘metafor’, and ‘meta’ R packages). All tests were two-sided and α was set at 0.05.

### Role of the funding source

Funders of the individual studies had no role in study design, data collection, data analysis, data interpretation, writing, or submission of the manuscript. Submission was decided by the corresponding author.

## Results

**Figure 1:**
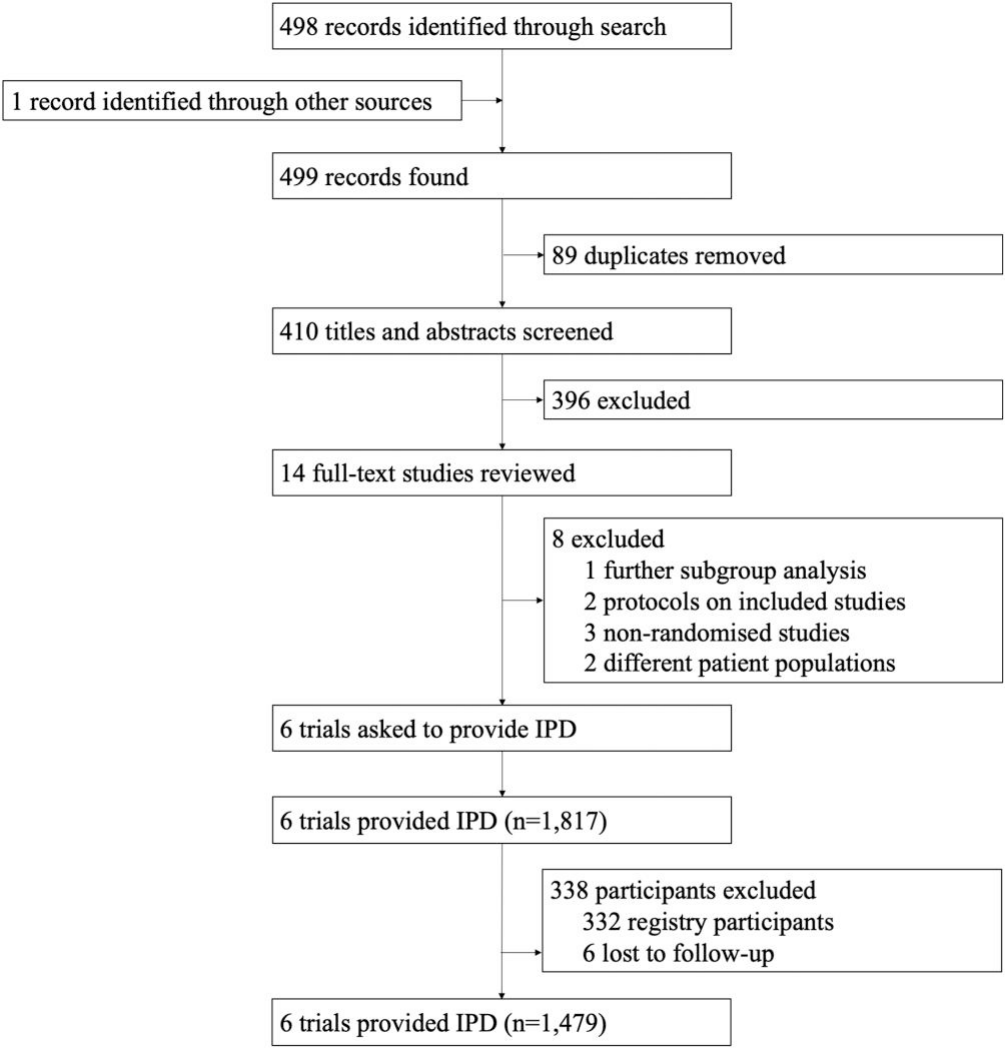
Study selection. Flow chart depicting studies screened and included in final analysis

Our search yielded 499 results from various literature sources. After removing duplicates, we screened 410 records by title and abstract. 14 studies were screened by full text for eligibility (see Supplementary data online, *Appendix* p4) and 6 studies were included in our meta-analysis (*Figure 1*). We received a positive response from the authors of all six eligible studies. The study design and quality of the included studies are summarized in the appendix (Supplementary data online, *Tables S2–S7* and *Appendix* pp 7–12). Overall, studies were assessed as low risk of bias (Online Supplementary data online, *Figure S1* and *Appendix* pp 21–22), accounting for the fact that blinding is not applicable given the nature of the intervention.

All six eligible trials contributed IPD and involved 1479 participants (*Table 1*). Trials were done between 2008 and 2021 in five countries. Median age was 84.0 years (interquartile range [IQR] 81.0–87.0), while 48% of participants were female (*Table 2*). Most participants had hypertension, with 29% suffering from diabetes mellitus at index hospitalization. In addition, 20% of the population had a previous PCI, with 35% having experienced a MI. Only 4.7% of participants were active smokers, while 43% had never smoked. The majority of patients received anti-platelets and beta blockers on discharge (*Table 3*), and all baseline demographic characteristics were well balanced between the randomization groups. As for randomization, 736 patients were randomized to initial invasive treatment and 743 patients were assigned to the initial conservative group (CG). A more detailed table with variables stratified by each individual trial is in the appendix (Supplementary data online, *Table S8* and *Appendix* pp 13–18).

**Table 1:** Key features of included trials

Trial	Enrolment	Population	Total Participants	Numbers of Participants		Median Timing of Angiography	Crossover from Conservative to PCI or CABG (%)
				Conservative Group	Routine Invasive Group		
Italian Elderly ACS Savonitto et al., 2012	January 2008—May 2010 Multi-centre RCT Italy	NSTEACS ≥ 75 years	313	159	154	1 day	23
After Eighty Tegn et al., 2016	December 2010—February 2014 Multi-centre RCT Norway	NSTEACS ≥ 80 years	457	228	229	1.4 days	-
MOSCA Sanchis et al., 2016	January 2012—March 2014 Multi-centre RCT Spain	NSTEMI ≥ 70 years	106	54	52	Not available	9
80+ Study Hirlekar et al., 2020	September 2009—September 2017 Multi-centre RCT Sweden	NSTEACS ≥ 80 years	186	93	93	Not available	4
RINCAL De Belder et al., 2021	May 2014—September 2018 Multi-centre RCT United Kingdom	NSTEMI ≥ 80 years	250	126	124	2 days	3
MOSCA-FRAIL Sanchis et al., 2023	July 2017—January 2021 Multi-centre RCT Spain	NSTEMI ≥ 70 years	167	83	84	Not available	10

**Table 2:** Patient characteristics

	Overall *n* = 1479	Treatment group	
		Conservative group *n* = 743	Invasive group *n* = 736
**Age (years)**	84.0 (81.0, 87.0)	84.0 (81.0, 87.0)	84.0 (81.0, 87.0)
**Sex (male)**	762 (52%)	394 (53%)	368 (50%)
**Smoking**			
Active smoker	69 (4.7%)	32 (4.3%)	37 (5.0%)
Ex-smoker	432 (29%)	226 (30%)	206 (28%)
Never smoker	630 (43%)	311 (42%)	319 (43%)
Missing values	348 (24%)	174 (23%)	174 (24%)
**Previous myocardial infarction**			
Yes	522 (35%)	269 (36%)	253 (34%)
Missing values	12 (0.8%)	4 (0.5%)	8 (1.1%)
**Previous CABG**			
Yes	189 (13%)	83 (11%)	106 (14%)
Missing values	9 (0.6%)	4 (0.5%)	5 (0.7%)
**Previous PCI**			
Yes	289 (20%)	151 (20%)	138 (19%)
Missing values	11 (0.7%)	3 (0.4%)	8 (1.1%)
**Hypertension**			
Yes	1057 (71%)	523 (70%)	534 (73%)
Missing values	8 (0.5%)	3 (0.4%)	5 (0.7%)
**Diabetes mellitus**			
Yes	423 (29%)	199 (27%)	224 (30%)
Missing values	7 (0.5%)	2 (0.3%)	5 (0.7%)
**Previous stroke**			
Yes	225 (15%)	114 (15%)	111 (15%)
Missing values	8 (0.5%)	3 (0.4%)	5 (0.7%)
**Previous peripheral arterial disease**			
Yes	125 (8.5%)	69 (9.3%)	56 (7.6%)
Missing values	322 (22%)	162 (22%)	160 (22%)
**Atrial fibrillation**			
Yes	216 (15%)	107 (14%)	109 (15%)
Missing values	366 (25%)	183 (25%)	183 (25%)
**Killip class**			
1	941 (64%)	471 (63%)	470 (64%)
2	257 (17%)	134 (18%)	123 (17%)
3	30 (2.0%)	12 (1.6%)	18 (2.4%)
4	5 (0.3%)	3 (0.4%)	2 (0.3%)
Missing values	246 (17%)	123 (17%)	123 (17%)

Median (IQR) or Frequency (%).

CABG, coronary artery bypass graft; PCI, percutaneous coronary intervention.

**Table 3:** Discharge medications

	Overall *n* = 1479	Treatment group	
		Conservative group *n* = 743	Invasive group *n* = 736
**Aspirin**			
Yes	1228 (83%)	620 (83%)	608 (83%)
Missing values	27 (1.8%)	12 (1.6%)	15 (2.0%)
**P2Y12 inhibitors**			
Yes	1206 (82%)	621 (84%)	585 (79%)
Missing values	25 (1.7%)	9 (1.2%)	16 (2.2%)
**Beta blockers**			
Yes	907 (61%)	464 (62%)	443 (60%)
Missing values	24 (1.6%)	9 (1.2%)	15 (2.0%)
**ACEi**			
Yes	697 (47%)	346 (47%)	351 (48%)
Missing values	28 (1.9%)	12 (1.6%)	16 (2.2%)
**ARBs**			
Yes	140 (9.5%)	76 (10%)	64 (8.7%)
Missing values	286 (19%)	141 (19%)	145 (20%)
**Statin**			
Yes	1181 (80%)	595 (80%)	586 (80%)
Missing values	29 (2.0%)	13 (1.7%)	16 (2.2%)
**Calcium channel blocker**			
Yes	255 (17%)	130 (17%)	125 (17%)
Missing values	287 (19%)	141 (19%)	146 (20%)
**Nitrates**			
Yes	621 (42%)	344 (46%)	277 (38%)
Missing values	289 (20%)	142 (19%)	147 (20%)
**Anticoagulation**			
Yes	203 (14%)	108 (15%)	95 (13%)
Missing values	122 (8.2%)	61 (8.2%)	61 (8.3%)

Median (IQR) or Frequency (%).

ACEi, angiotensin-converting-enzyme inhibitors; ARBs, angiotensin II receptor blockers; P2Y12 inhibitor, purinergic receptor inhibitor antiplatelet.

**Figure 2:**
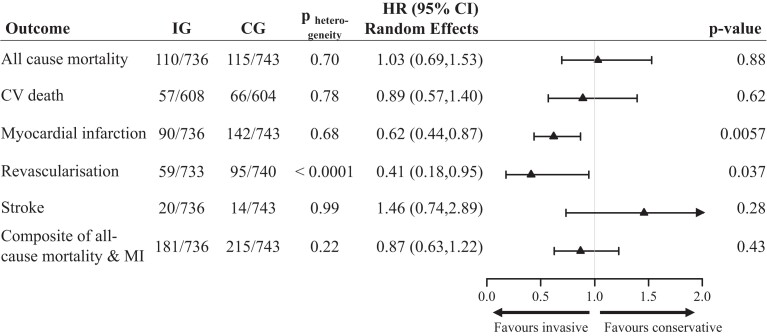
Adverse outcomes 1 year post-randomization. Plots presenting hazard ratios and 95% confidence intervals for the comparison of participants in the invasive group vs. the conservative group using random-effects Cox models to adjust for within-study clustering. The numbers in the invasive group and conservative group columns represent the number of events/total number of participants in each group. Denominators are different for cardiovascular death, due to missingness of this outcome in one study, and for the revascularization endpoint, because six participants were missing follow-up time values. The composite endpoint includes all-cause mortality and/or myocardial infarction

Our primary synthesis revealed no significant treatment difference for the composite endpoint of all-cause mortality and MI during a 1-year follow-up period, with an incidence of 24.5% in the early invasive management group and 28.9% in the conservative management group and an unadjusted HR of 0.87 (95% CI 0.63–1.22; *P* = .43) using a random-effects model and 0.82 (95% CI 0.67–1.00; *P* = .0503) using a fixed-effect model (*Figure 2* and Supplementary data online, *Figure S2* and *Appendix* p23). MI had a significantly lower incidence in the invasive group (IG) compared to the conservative management group (12.2% vs. 19.1%), indicating that early invasive management in older persons substantially reduces the hazard of a further MI within the first year with HR from random-effects model 0.62 (95% CI 0.44–0.87; *P* = .006) and HR from fixed-effect model 0.61 (95% CI 0.47–0.79; *P* = .0002). In addition, the need for repeat revascularization within 365 days of a NSTEACS was substantially reduced for older persons receiving early invasive therapy as compared to persons treated initially with medical therapy with HR from random-effects model 0.41 (95% CI 0.18–0.95; *P* = .037), and HR from fixed-effect model 0.60 (95% CI 0.43–0.83; *P* = .002). The endpoint of cardiovascular death was not reported in one trial and there was no significant difference in the hazard of cardiovascular mortality between participants in the invasive and conservative groups (HR from random-effects model 0.89, 95% CI 0.57–1.40; *P* = .62). Finally, 34 participants suffered a stroke within the first year (HR from random-effects model 1.46, 95% CI 0.74–2.89; *P* = .28). Applying a fixed-effect approach to our primary analysis reproduced similar results except from the composite endpoint, for which the fixed-effect Cox model yielded an almost significant result in favour of the invasive strategy (Supplementary data online, *Figure S2* and *Appendix* p 23). We further present forest plots using a two-stage meta-analysis approach without adjustment for within-trial clustering for the composite, MI, and revascularization endpoints in the appendix (Supplementary data online, *Figure S3* and *Appendix* pp24–25). Heterogeneity tests for studied outcomes (Supplementary data online, *Figure S3* and *Appendix* pp 24–25) showed no significance apart from the revascularization endpoint, which was mainly driven by the high HR for that endpoint in the Italian Elderly ACS study.

We repeated our primary analyses utilizing random-effects and fixed-effect Cox models adjusted for age, sex, hypertension, and diabetes (Supplementary data online, *Figure S4* and *Appendix* p26). We observed no particular change in the direction of results apart from the composite endpoint which reached significance in the adjusted fixed-effect model (aHR 0.82, 95% CI 0.67–1.00; *P* = .048), indicating a potential superiority of early invasive strategy as compared to conservative management.

**Figure 3:**
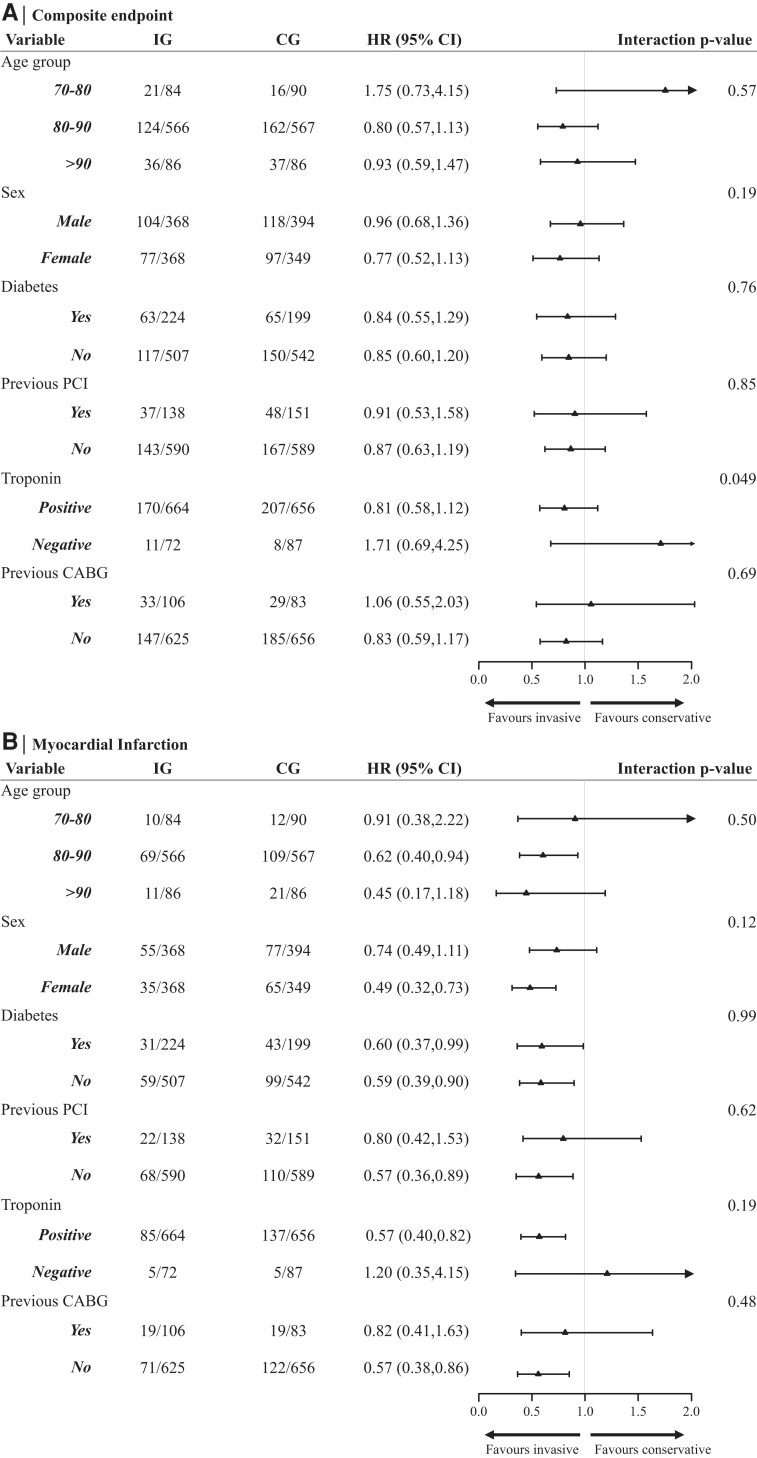
Subgroup analyses. Hazard ratios for subgroup analyses based on age groups, sex, diabetes mellitus, history of previous percutaneous coronary intervention, troponin levels, and history of previous coronary artery bypass graft surgery for the composite endpoint (*A*) and myocardial infarction (*B*) 1 year post-randomization. Random-effects Cox models were fitted to adjust for within-study clustering. *P*-values presented are for interaction terms. *P*-value for the age group variable represents the trend *P*-value. The numbers in the invasive group and conservative group columns represent the number of events/total number of participants in each group. The composite endpoint includes all-cause mortality and/or myocardial infarction

In subgroup analyses, we *a priori* assessed the effect of different age groups, sex, and diabetes on the primary and MI endpoints (*Figure 3*) and also a post-hoc sub-group analysis was done for elevated troponin levels and previous CABG. All tests for interaction showed no significant difference, except for the interaction of troponin status for the primary endpoint (*P* = .049). Peri-procedural MI (PMI) was inconsistently reported across studies. In order to perform sensitivity analyses excluding PMI, which may present with a different prognosis compared to spontaneous MI, we consistently applied the Type 4a universal definition of MI across all studies, excluding all patients from the IG with MI 48 h post-randomization. As for the CG, we identified only two patients that had crossover to angiography and revascularization, presenting with MI <48 post-randomization (Supplementary data online, *Figure S5* and *Appendix* p27). We excluded those patients as well from our sensitivity analyses to avoid the introduction of bias, as we had no access to exact timing of procedures and events, therefore, we were unable to establish whether those MIs were PMI. We observed a stronger treatment effect after exclusion of PMI, with a HR from random-effects model 0.56 (95% CI 0.40–0.78; *P* < .0001) (Supplementary data online, *Figure S5* and *Appendix* p 27).

In further analysis for visual purposes only, the rate of patients from the CG needing coronary angiography and receiving revascularization during the index hospitalization did not appear to have a clear influence on the effect size of the primary endpoint (Supplementary data online, *Figure S6* and *Appendix* p28). Further, in sensitivity analyses excluding patients from the CG undergoing coronary angiography, we observed similar results for all endpoints, apart from revascularisation, which lost its significance with HR 0.49 (95% CI 0.21–1.13; *P* = .094) (Online Supplementary data online, *Figure S7* and *Appendix* p 29). In addition, we visually observed no effect of publication year on the composite endpoint (Online Supplementary data online, *Figure S8* and *Appendix* p 30) or any publication bias as evidenced by a balanced Funnel plot (Supplementary data online, *Figure S9* and *Appendix* p 31).

Bleeding events were reported qualitatively due to the six trials using different endpoint definitions (Supplementary data online, *Table S9* and *Appendix* p 19). Generally bleeding rates were low and only one trial reported a significant difference between routine invasive and conservative groups. Similarly, data on the use of radial access and drug-eluting stents were not available on an IPD level for all trials. These rates have been summarized (Supplementary data online, *Table S10* and *Appendix* p 20).

## Discussion

Using IPD, this meta-analysis shows that the risk of MI and unplanned revascularization is lower in older patients with NSTEACS treated with a routine invasive strategy compared to a conservative medical approach. The risk of the primary composite endpoint showed only weaker evidence of a potentially lower risk for an invasive strategy and there was no evident difference for all-cause mortality, cardiovascular death, and stroke (Structured Graphical Abstract).

Older patients presenting with ACS in clinical practice today are a heterogeneous group with characteristics such as frailty, multi-morbidity, and cognition influencing outcomes beyond just the remit of chronological age.^20–23^ They frequently have complex coronary artery lesions and present with competing risks of altered physiology, co-morbidities, and vulnerability to both ischaemic outcomes and bleeding events.^23–25^ International treatment guidelines recommend applying the same interventional strategies in older patients as for younger patients; that is to offer routine coronary angiography with revascularization if clinically indicated in patients identified as high risk of adverse outcomes based on risk scores.^5,26^ However, age over 75 years is the strongest negative predictor of receiving routine invasive care, with frail or co-morbid patients less likely to receive coronary angiography.^8,21,27^ Currently the risk-benefit ratio of routine invasive care in older patients is unclear given the paucity of evidence; due to the under-representation of this patient group in previous studies and recent trials being underpowered and producing inconsistent findings.^9^

The current study is the first IPD meta-analysis including all available trials that specifically enrolled older patients with NSTEACS. We included 1479 patients with a mean age of 84 years. The last substantial collaborative meta-analysis was a pooled subgroup analysis of the FRISC-II, ICTUS, and RITA-3 (FIR) trials from over two decades ago.^12^ These studies did not meet our pre-specified inclusion criteria, as they did not specifically include patients over the age of 70. Consistent application of inclusion criteria helps maintain the homogeneity of the included studies, ensuring that the pooled results accurately reflect the targeted research question, minimizing the risk of post-hoc decisions that could introduce bias. In addition, the oldest age category in the FIR dataset included 839 participants over the age of 75 years with a mean age of 76 years and fewer co-morbidities than the cohort we studied.

We found no significant difference in the risk of all-cause mortality or cardiovascular death between those receiving routine invasive care or conservative management strategies. This could be related to the short follow-up restricted to 1 year or the hetergogeneity of the population studied. Generally, observational studies have shown reductions in mortality rates with a routine invasive approach.^28,29^ However, these results are limited by selection bias and confounding by indication. No dedicated trial yet has identified an all-cause mortality benefit from routine invasive care in this patient group, raising a reasonable question around the utility of mortality as an endpoint in clinical trials of older people. There was no statistically significant difference in the hazard for our primary endpoint using random-effects Cox modelling, but the hazard of all-cause mortality and reinfarction did reach borderline significance with fixed-effect Cox modelling. Although the FIR meta-analysis and subgroup analysis of the TACTICS-TIMI 18 trial reported reductions in their similar composite endpoints in older patients treated with a routine invasive approach,^11,12^ these findings were driven by significantly lower rates of MI only—consistent with our results. All trials reported no difference in the hazard for stroke and this is consistent with previous registry data. We also reported a reduction in the need for urgent revascularization following a routine invasive strategy compared to a conservative approach. Older studies have not evaluated this outcome but a recent, large cohort study using propensity scores and inverse probability of treatment weighting also demonstrated a similar reduction in the hazard of this endpoint.^29^ This study using IPD demonstrates significant clinical benefits from a routine invasive management strategy, similar to subgroup findings of the FIR pooled analysis but in a considerably older and more co-morbid patient group receiving contemporary invasive care.

In a pre-specified subgroup analysis, we found no convincing evidence of effect modified. The After Eighty trial reported an attenuation of the efficacy of an invasive strategy with increasing age.^14^ In this meta-analysis, as the ≥90 age group comprised just 172 participants there is insufficient evidence to draw definitive conclusions. We have previously shown that in older adults with invasive treatment of NSTEACS, provision of guideline-directed care resulted in similar long-term outcomes between males and females.^30^ We observed more convincing evidence of clinical benefit from a routine invasive approach over a conservative strategy in older patients with elevated troponin levels. Patients with high admission troponin levels had a reduction in hazard for MI at 1 year by 40% if treated invasively, an effect which was reversed in patients with normal troponin levels.

Concerns regarding bleeding and its prognostic association with adverse long-term outcomes influence benefit-risk management decisions in older patients.^31^ Bleeding rates could not be assessed quantitatively in our study, but the majority of included trials reported no evidence of an increased risk of major bleeding. Trials done many years ago reported raised bleeding risk with invasive management of NSTEACS in older adults but in the era of radial access, drug-eluting stents, and appropriate antiplatelet strategies this risk is now attenuated. Indeed, no differences in bleeding rates between invasively and conservatively managed older patients were detected in the SENIOR-NSTEMI study.^29^ Notably, use of radial access and drug-eluting stents in these six trials was high, whilst bleeding rates were relatively low and comparable with recent studies investigating contemporary revascularization approaches in older patients at high bleeding risk.^32–34^

We conducted an intention-to-treat analysis, with crossover from the conservative arm to coronary angiography (crossover ranging from 3% to 23%, *Table 1*) according to clinical indication set out by the individual trials. An exploratory visualization demonstrated that the crossover rate of revascularization during the index hospitalization in patients from the CG did not appear to influence the hazard of the primary composite endpoint. Older patients may derive a mortality benefit from early coronary angiography, compared to a delayed approach.^35^ The timing of coronary angiography in older patients with NSTEACS may impact on clinical outcomes but unfortunately data on this variable were not available in all trials.

The ongoing large scale (*n* = 1518) British Heart Foundation SENIOR-RITA randomized trial (NCT03052036) aims to provide more robust evidence regarding the benefit-risk profile of a routine invasive strategy in this patient group. It has broad eligibility criteria, and will also evaluate frailty, multi-morbidity, and health-related quality of life.

### Strengths and limitations

This is the first meta-analysis using IPD comparing a routine invasive strategy with a conservative approach in older patients with NSTEACS. All trials provided data, reflecting contemporary interventional management and the heterogeneous older patient population encountered in clinical practice today, as well as overcoming challenges facing previous meta-analyses of aggregate data such as differing definitions of composite endpoints and inconsistent follow-up periods. Herein, we present a synthesis of evidence that comments on the quality of existing trials using metrics of heterogeneity and the Cochrane Collaboration’s Risk of Bias 2 tool, and unifies diverse sources of data, increasing statistical power, and offering a more comprehensive perspective on the efficacy of invasive vs. conservative management of NSTEACS among older adults across a broader range of populations and settings.

There are limitations to this study. First, we were unable to quantitatively evaluate bleeding as an outcome due to differing definitions of this endpoint within the constituent trials. However, bleeding rates were low and comparable with other trials investigating bleeding-reduction strategies in this patient group. Second, the timing of angiography was not standardized across all six trials (missing in two studies), but this reflects the provision of PCI facilities across several regions of the developed world. Third, there was a lack of frailty assessment, with only the MOSCA-FRAIL trial providing such information. Frailty is prevalent in older patients with NSTEACS and may impact on any benefits derived from coronary angiography and revascularization. Also, no data on angiographic complexity of coronary artery disease was available. Further, the majority of our population was aged between 80 and 90 years old, making our conclusion most relevant to this age group. Censoring was limited to 1 year, which was selected as this was the minimum follow-up period across all studies included. In addition, given the advanced age and co-morbidity of the population studied, we feel that 1-year outcomes are the most relevant in assessing superiority of one management approach vs. the other, as these would have the most important impact on patient life expectancy, prognosis, and quality of life. Blinding to the intervention was also not possible due to the nature of the intervention. This was common to all trials but remains a limitation nonetheless. All outcomes presented in our main manuscript were analysed using random-effects models, which are often considered preferable to fixed-effect models as they account for inherent variability and heterogeneity among studies, providing a more realistic and generalizable estimation by allowing for the possibility that true treatment effects may vary across studies due to diverse populations, methodologies, or other underlying factors.^36^ However, the relative means of the two meta-analyses approaches remain controversial, and so we provide a balanced account by presenting fixed-effect models in the appendix. Finally, given the lack of data on the exact timings of procedures, we recognize a limitation in our PMI sensitivity analyses in identifying all possible PMIs. However, this is the best data mining attempt we can present without introducing bias or making undue assumptions.

## Conclusions

This is the first IPD meta-analysis comparing invasive vs. conservative management in older patients with NSTEACS. A routine invasive strategy was associated with a substantial reduction in the risk of reinfarction and urgent revascularization. However, no significant difference was observed for all-cause mortality or the composite of all-cause mortality and MI at 1 year.

In the absence of robust evidence from an adequately powered randomized controlled trial, this study strengthens the evidence base supporting an interventional approach in this patient group, thus serving to better inform a more balanced approach to individualized treatment strategy.

## Supplementary Material

ehae151_Supplementary_Data

## Declarations

### Data Availability

Individual participant-level data used for this report are not publicly available, because they contain protected patient health information. Requests for data access should be directed to the corresponding author via email.

### Pre-registered Clinical Trial Number

This meta-analysis is registered with PROSPERO (CRD42023379819).
