# Supplementary material for: Invasive vs. conservative management of older patients with non-ST-elevation acute coronary syndrome: individual patient data meta-analysis
Source: Eur Heart J. 2024 Apr 10;45(23):2052–62. doi: 10.1093/eurheartj/ehae151 (PMC11177715; doi:10.1093/eurheartj/ehae151)
Supplement: ehae151_Supplementary_Data [file ehae151_supplementary_data.docx]

**APPENDIX**

**Invasive versus initial conservative management of older patients with non-ST elevation myocardial infarction or unstable angina:
A systematic review and individual patient data meta-analysis**

Christos P. Kotanidis DPhil, Gregory B. Mills MBBS MRes, Professor Bjørn Bendz MD, Erlend S. Berg MD, Professor David Hildick-Smith MD, Geir Hirlekar MD, Dejan Milasinovic MD, Nuccia Morici MD, Aung Myat MD, Nicolai Tegn MD, Professor Juan Sanchis MD, Stefano Savonitto MD, Professor Stefano De Servi MD, Professor Keith Fox MB ChB, Professor Stuart Pocock PhD, Professor Vijay Kunadian MD

**Table of contents**

[Supplementary Methods 3](#_Toc155632144)

[List of studies excluded at full-text screening stage, with brief explanations 4](#_Toc155632145)

[Online Table 1 | Endpoint definitions across trials 6](#_Toc155632146)

[Online Table 2 | Key Information: Savonitto et al., 2012 (Italian Elderly ACS) 7](#_Toc155632147)

[Online Table 3 | Key Information: Tegn et al., 2016 (After Eighty) 8](#_Toc155632148)

[Online Table 4 | Key Information: Sanchis et al., 2016 (MOSCA) 9](#_Toc155632149)

[Online Table 5 | Key Information: Hirlekar et al., 2020 (80+ Study) 10](#_Toc155632150)

[Online Table 6 | Key Information: De Belder et al., 2021 (RINCAL) 11](#_Toc155632151)

[Online Table 7 | Key Information: Sanchis et al., 2023 (MOSCA-FRAIL) 12](#_Toc155632152)

[Online Table 8 | Demographics of participants across trials included in analysis 13](#_Toc155632153)

[Online Table 9 | Bleeding Events 19](#_Toc155632154)

[Online Table 10 | Use of Radial Access and Drug-Eluting Stents 20](#_Toc155632155)

[Online Figure 1 | Risk of bias assessment 21](#_Toc155632156)

[Online Figure 2 | Adverse outcomes one year post randomisation. 23](#_Toc155632157)

[Online Figure 3 | Random effects forest plots for individual studies 25](#_Toc155632158)

[Online Figure 4 | Sensitivity analyses for adverse outcomes one year post randomisation 26](#_Toc155632159)

[Online Figure 5 | Sensitivity analysis excluding peri-procedural MI 27](#_Toc155632160)

[Online Figure 6 | Meta-regression analysis of crossover rates 28](#_Toc155632161)

[Online Figure 7 | Adverse outcomes one year post randomisation excluding crossover patients. 29](#_Toc155632162)

[Online Figure 8 | Meta-regression analysis of publication year 30](#_Toc155632163)

[Online Figure 9 | Funnel plot. 31](#_Toc155632164)

[PRISMA-IPD Checklist 32](#_Toc155632165)

# Supplementary Methods

***Search algorithm***

The following terms were used with no restriction for language: (nsteacs OR NSTEMI) AND

((angioplasty, transluminal, percutaneous coronary[mh]) OR pci OR angioplasty OR coronary artery bypass OR revascularisation OR invasive) AND (care standard[mh] OR “optical medical therap*” OR “optical medical treatment” OR “optical medical management” OR “OMT” OR “conservative management” OR “conservative treatment” OR “conservative therap*”) AND (aged[mh] OR aged, 80 and over[mh]).

***Data cleaning***

In the Eighty+ study one participant’s inclusion date was missing the day value, which was arbitrarily replaced with the first day of the inclusion month provided. In addition, we have discovered a discrepancy in the numbers reported for the “sex” variable in the After Eighty study between the dataset received and data on the public domain. We contacted the authors, who kindly agreed to resolve the issue, apparently attributable to an eCRF exporting error. The correct male and female distribution numbers are the ones reported within this article, rather than the original publication.

# List of studies excluded at full-text screening stage, with brief explanations

Gündoğmuş PD, Ölçü EB, Öz A, Tanboğa İH, Orhan AL. The effects of percutaneous coronary intervention on mortality in elderly patients with non-ST-segment elevation myocardial infarction undergoing coronary angiography. Scott Med J. 2020 Aug;65(3):81-88. doi: 10.1177/0036933020919931. PMID: 32772677.

- *Excluded as it was an observational study.*

Hoedemaker NPG, Damman P, Woudstra P, Hirsch A, Windhausen F, Tijssen JGP, de Winter RJ; ICTUS Investigators. Early Invasive Versus Selective Strategy for Non-ST-Segment Elevation Acute Coronary Syndrome: The ICTUS Trial. J Am Coll Cardiol. 2017 Apr 18;69(15):1883-1893. doi: 10.1016/j.jacc.2017.02.023. PMID: 28408018.

- *Excluded as it investigated a different patient population (patients of all ages) rather than older patients with NSTEACS specifically.*

Lee MMY, Petrie MC, Rocchiccioli P, Simpson J, Jackson CE, Corcoran DS, Mangion K, Brown A, Cialdella P, Sidik NP, McEntegart MB, Shaukat A, Rae AP, Hood SHM, Peat EE, Findlay IN, Murphy CL, Cormack AJ, Bukov NB, Balachandran KP, Oldroyd KG, Ford I, Wu O, McConnachie A, Barry SJE, Berry C; CABG-ACS Investigators. Invasive Versus Medical Management in Patients With Prior Coronary Artery Bypass Surgery With a Non-ST Segment Elevation Acute Coronary Syndrome. Circ Cardiovasc Interv. 2019 Aug;12(8):e007830. doi: 10.1161/CIRCINTERVENTIONS.119.007830. Epub 2019 Jul 31. PMID: 31362541; PMCID: PMC7664981.

- *Excluded as it investigated a different patient population (patients of all ages with prior CABG) rather than older patients with NSTEACS specifically.*

Sampaio F, Roberto J, Cruz F, Mateus P, Gonçalves C, Gonçalves M, Simões L, Gama V. Two different invasive versus noninvasive strategies in the management of non-ST elevation acute coronary syndromes. Rev Port Cardiol. 2007 Feb;26(2):117-24. English, Portuguese. PMID: 17479707.

- *Excluded as it was a retrospective study investigating the timing of an invasive strategy in younger patients.*

Sanchis J, Ariza-Solé A, Abu-Assi E, Alegre O, Alfonso F, Barrabés JA, Baz JA, Carol A, Díez Villanueva P, García Del Blanco B, Elízaga J, Fernandez E, García Del Egido A, García Picard J, Gómez Blázquez I, Gómez Hospital JA, Hernández-Antolín R, Llibre C, Marín F, Martí Sánchez D, Martín R, Martínez Sellés M, Miñana G, Morales Gallardo MJ, Núñez J, Pérez de Prado A, Pinar E, Sanmartín M, Sionis A, Villa A, Marrugat J, Bueno H. Invasive Versus Conservative Strategy in Frail Patients With NSTEMI: The MOSCA-FRAIL Clinical Trial Study Design. Rev Esp Cardiol (Engl Ed). 2019 Feb;72(2):154-159. English, Spanish. doi: 10.1016/j.rec.2018.02.007. Epub 2018 Mar 7. PMID: 29525724.

- *Excluded as it was the published protocol for the MOSCA randomised trial (included in this IPD meta-analysis).*

Savonitto S, De Servi S, Petronio AS, Bolognese L, Cavallini C, Greco C, Indolfi C, Visconti LO, Piscione F, Ambrosio G, Galvani M, Marzocchi A, Santilli I, Steffenino G, Maseri A. Early aggressive vs. initially conservative treatment in elderly patients with non-ST-elevation acute coronary syndrome: the Italian Elderly ACS study. J Cardiovasc Med (Hagerstown). 2008 Mar;9(3):217-26. doi: 10.2459/JCM.0b013e3282f7c8df. PMID: 18301136.

- *Excluded as it was the published protocol for the Italian Elderly ACS randomised trial (included in this IPD meta-analysis).*

Tegn N, Abdelnoor M, Aaberge L, Hylen Ranhoff A, Endresen K, Gjertsen E, Skårdal R, Gullestad L, Bendz B; After Eighty study investigators. Health-related quality of life in older patients with acute coronary syndrome randomised to an invasive or conservative strategy. The After Eighty randomised controlled trial. Age Ageing. 2018 Jan 1;47(1):42-47. doi: 10.1093/ageing/afx121. PMID: 28985265.

- *Excluded as it was a subgroup analysis of the After Eighty study (already included in this IPD meta-analysis).*

Yilmaz S, Adali MK, Kilic O, Til A, Yaylali YT. Effect of invasive strategy on long-term mortality in elderly patients presenting with acute coronary syndrome. Cardiovasc J Afr. 2020 Sep/Oct;31(5):252-256. doi: 10.5830/CVJA-2020-011. Epub 2020 Jun 22. PMID: 32628742; PMCID: PMC8762820.

- *Excluded as it was a retrospective cross-sectional study.*

# Online Table 1 | Endpoint definitions across trials

| **Study** | **Primary composite endpoint** | **Non-fatal MI** | **Stroke** | **Bleeding** |
| --- | --- | --- | --- | --- |
| Savonitto et al., 2012  Italian Elderly ACS | All-cause mortality, reinfarction, stroke and readmission for cardiovascular causes or severe bleeding | New cardiac symptoms with ECG evidence and elevated cardiac biomarkers > 99^th^ centile. | New focal neurological deficit with duration > 24 hours confirmed by appropriate imaging, with classification as ‘disabling’ or non-disabling. | ‘Severe’ bleeding leading to hospital admission, including BARC ≥2 criteria. |
| Tegn et al., 2016  After Eighty | All-cause mortality, reinfarction, urgent revascularisation and stroke | New cardiac symptoms with troponin > 99^th^ percentile. Periprocedural MI (type 4a: within 48 hours after index procedure) defined as rise in cardiac biomarkers beyond three times the 99^th^ centile | New focal neurological deficit of vascular origin lasting > 24 hours. | ‘Major’ or ‘minor’ according to TIMI criteria. |
| Sanchis et al., 2016  MOSCA | All-cause mortality, reinfarction and readmission for cardiac causes (revascularisation or heart failure) | New cardiac symptoms with troponin elevation. Periprocedural MI defined as troponin elevation beyond five times the 99^th^ centile (after PCI) or ten times the 99^th^ centile (after CABG) 12 hours after revascularization. |  | TIMI ≥2 criteria. |
| Hirelekar et al., 2020  80+ Study | All-cause mortality, reinfarction, urgent revascularisation, stroke and recurrent hospitalisation for cardiac reasons (heart failure or new onset atrial fibrillation) | New cardiac symptoms with troponin > 99^th^ percentile. Periprocedural MI defined as rise in cardiac biomarkers beyond three times the 99^th^ centile. | Cerebral ischaemic events including transient ischaemic attack. | ‘Major’ or ‘minor’ according to TIMI criteria. |
| De Belder et al., 2021  RINCAL | All-cause mortality and reinfarction | New cardiac symptoms with troponin > 99^th^ percentile. Periprocedural MI as per the third universal definition of MI. | New focal neurological deficit with duration > 24 hours confirmed by a neurologist and appropriate imaging. | BARC ≥3B criteria. |
| Sanchis et al., 2023  MOSCA-FRAIL | Cardiac death, reinfarction and post-discharge revascularisation.  Number of days alive and out of hospital (encompassing mortality and hospitalisations). | New chest pain and troponin elevation consistent with most recent universal definition of myocardial infarction. |  | Any bleeding requiring hospitalisation. |

# Online Table 2 | Key Information: Savonitto et al., 2012 (Italian Elderly ACS)

| **Principal Investigator** | **S. Savonitto and S. De Servi** |
| --- | --- |
| **Enrolment** | **Italy, January 2008 – May 2010** |
| **Study Design** | **Randomised controlled study**  ***‘Invasive’***. Coronary angiography within 72 hours and, when indicated, coronary revascularization by either PCI or CABG according to coronary anatomy, patient preference, and local skills.  ***‘Conservative’*.** Patients were managed with guideline-directed medical therapy, and coronary angiography during index hospital stay was allowed in the case of refractory ischemia, myocardial (re)infarction, heart failure of ischemic origin, or malignant ventricular arrhythmias. |
| **Inclusion Criteria** | Eligible were patients with NSTEACS aged 75 years or greater, with cardiac ischemic symptoms at rest within 48 hr before randomization, together with ischemic ECG changes and/or elevated levels of either troponin or CK-MB. |
| **Exclusion Criteria** | Excluded were patients with secondary causes of myocardial ischemia, ongoing myocardial ischemia or heart failure despite optimized therapy, PCI or CABG within 30 days before randomization, serum creatinine greater than 2.5 mg/dl, a cerebrovascular accident within the previous month, recent transfusions, gastrointestinal or genitourinary bleeding within 6 weeks before randomization, platelet count less than 90,000 cells/micro-l, ongoing oral anticoagulation, severe obstructive lung disease, malignancy, or neurological deficit limiting follow-up. |
| **Follow up visits** | 30 days, 6 months and 12 months post-randomisation |
| **Participants** | 313 patients with NSTEACS aged ≥ 75 years – 190 troponin-positive patients and 123 troponin-negative patients |
| **Outcomes** | ***Primary***: composite of all-cause mortality, non-fatal myocardial infarction, disabling stroke and repeat hospital stay for cardiovascular causes or severe bleeding at 12 months.  ***Secondary***: all-cause mortality, cardiac & non-cardiac death, myocardial infarction, cardiac death and myocardial infarction, disabling stroke, repeat hospital stay for cardiovascular causes or severe bleeding at 12 months. |
| **Ethical Review** | The study was approved by the ethics committees of the participating hospitals. |
| **Overall bias assessment** | Low risk |

# Online Table 3 | Key Information: Tegn et al., 2016 (After Eighty)

| **Principal Investigator** | **B. Bendz** |
| --- | --- |
| **Enrolment** | **Norway, December 2010 – February 2014** |
| **Study Design** | **Randomised controlled study**  ***‘Invasive’***. Early coronary angiography with immediate assessment for ad-hoc percutaneous coronary intervention, coronary artery bypass graft, in addition to optimum medical treatment.  ***‘Conservative’*.** Optimum medical treatment alone, according to existing guidelines. |
| **Inclusion Criteria** | The trial enrolled consecutive consenting clinically stable patients aged 80 years or older, with NSTEMI or unstable angina, with or without ST-segment depression on electrocardiogram (ECG), and with normal or raised blood concentration of troponin T or I. |
| **Exclusion Criteria** | Patients were ineligible if they were clinically unstable with continuing chest pain or other ischaemic symptoms or signs, cardiogenic shock, continuing bleeding problems, or short life expectancy (<12 months) because of serious comorbidity (such as chronic obstructive pulmonary disease, disseminated malignant disease, or other reasons). Substantial mental disorder, including severe dementia or any disorder that interfered with a patient’s ability to comply with the protocol, was also an exclusion criterion. |
| **Follow up visits** | Continuous assessment through feedback by phone and written reports from hospitals, and adverse events from the data and safety monitoring board. |
| **Participants** | 457 patients with NSTEACS aged ≥ 80 years – 425 troponin-positive patients and 32 troponin-negatives patients |
| **Outcomes** | ***Primary***: composite of myocardial infarction, need for urgent revascularisation, stroke and death at median 1.53 years.  ***Secondary***: all-cause mortality, myocardial infarction, need for urgent revascularisation, stroke and bleeding at median 1.53 years. |
| **Ethical Review** | The study was approved by the regional board of research ethics. |
| **Overall bias assessment** | Low risk |

# Online Table 4 | Key Information: Sanchis et al., 2016 (MOSCA)

| **Principal Investigator** | **J. Nunez** |
| --- | --- |
| **Enrolement** | **Spain, January 2012 – March 2014** |
| **Study Design** | **Randomised controlled study**  ***‘Invasive’***. The invasive management group received routine cardiac catheterization within 72 h of admission, in addition to medical treatment optimised according to guidelines.  ***‘Conservative’*.** Patients underwent only medical treatment optimised according to guidelines, although cardiac catheterization was allowed in the case of poor in-hospital outcome due to recurrent ischemia or heart failure after admission, or in the case of a positive pre-discharge non-invasive stress test. |
| **Inclusion Criteria** | Patients were eligible for inclusion if they fulfilled all three of the following criteria:  1) Non-STEMI, defined by acute chest pain, non- STE electrocardiogram and troponin elevation (according to the local lab- oratory troponin assay);  2) age ≥ 70 years;  3) significant comorbidities defined by, at least, two of the following: renal failure (glomerular filtration rate < 45 ml/min/m2 at admission), neurological disease with a residual deficit (Rankin scale > 1), documented peripheral artery disease (ankle/brachial index < 0.9), dementia (≥ 3 mistakes in Pfeiffer's test), chronic pulmonary disease (forced expiratory volume in one second <50% or need of ambulatory oxygen therapy) or anaemia (haemoglobin <11 g/dl). |
| **Exclusion Criteria** | The exclusion criteria were the following:  1) Dynamic ST- segment changes suggestive of ischemia, because it was deemed unethical to deny invasive management to these patients (fixed ST- depression, however, was not an exclusion criterion);  2) prior known non-revascularizable coronary artery disease;  3) concomitant heart disease different than ischemic heart disease;  4) life expectancy < 1 year. |
| **Follow up visits** | Detailed clinical and treatment data were collected on hospitalisations. |
| **Participants** | 106 patients with NSTEMI aged ≥ 70 years |
| **Outcomes** | ***Primary***: composite of all-cause mortality, recurrent myocardial infarction and readmission for cardiac cause (revascularisation or heart failure) at median 2.5 years.  ***Secondary***: all-cause mortality, reinfarction, death and reinfarction and revascularisation, and bleeding at median 2.5 years. |
| **Ethical Review** | The study was approved by the regional board of research ethics. |
| **Overall bias assessment** | Low risk |

# Online Table 5 | Key Information: Hirlekar et al., 2020 (80+ Study)

| **Principal Investigator** | **G. Hirlekar** |
| --- | --- |
| **Enrolment** | **Sweden, September 2009 – September 2017** |
| **Study Design** | **Randomised controlled study**  ***‘Invasive’***. Coronary angiography and, if appropriate, revascularization with PCI or coronary artery bypass grafting (CABG), and optimal medical treatment as per ESC guidelines.  ***‘Conservative’*.** Optimal medical treatment without coronary angiography. Patients were able to undergo coronary angiography if refractory chest pain, hemodynamic instability, heart failure, or life-threatening cardiac arrhythmias, and then revascularized if appropriate. |
| **Inclusion Criteria** | The inclusion criteria were:  (1) 80 years or older;  (2) NSTE-ACS with ischemic symptoms (chest pain) lasting over 10min in the previous 72h;  (3) ischemic ST-segment depression >1 mm and/or elevated troponin I, troponin T, or CK-MB. |
| **Exclusion Criteria** | The exclusion criteria were: (1) PCI within 30 days prior to randomization; (2) ongoing active internal bleeding; (3) ST-segment elevation of >1mm in two contiguous leads on ECG; (4) enrolled in another study that has not completed follow-up; (5) known allergy to aspirin or P2Y12 antagonists; (5) severe dementia; (6) expected limited 1-year survival due to other diseases; and/ or (7) unwillingness to participate or expected problems with compliance. |
| **Follow up visits** | All patients were assessed for events by telephone and medical records. |
| **Participants** | 186 patients with NSTEACS aged ≥ 80 years – 180 troponin-positive patients and 6 troponin-negative patients |
| **Outcomes** | ***Primary***: composite of all-cause mortality, myocardial infarction, urgent revascularisation, stroke and recurrent hospitalisation for cardiac reasons at 12 months.  ***Secondary***: all-cause mortality, myocardial infarction, urgent revascularisation, stroke, recurrent hospitalisation for cardiac reasons, death and myocardial infarction, and bleeding at 12 months. |
| **Ethical Review** | The local ethics committee approved the study. |
| **Overall bias assessment** | Low risk |

# Online Table 6 | Key Information: De Belder et al., 2021 (RINCAL)

| **Principal Investigator** | **D. Hildick-Smith** |
| --- | --- |
| **Enrolment** | **United Kingdom, May 2014 – September 2018** |
| **Study Design** | **Randomised controlled study**  ***‘Invasive’***. Coronary angiography was conducted with physiological determination of intermediate lesions by fractional flow reserve or instantaneous wave-free ratio at the operator’s discretion. Significant coronary lesions were treated with *ad hoc* percutaneous coronary intervention (PCI), coronary artery bypass graft (CABG) surgery or referred directly for further discussion by a Heart Team. Coronary anatomy not requiring or amenable to intervention was subsequently managed with optimal medical therapy.  ***‘Conservative’*.** Optimal medical treatment alone according to guidelines. Patients were permitted to have diagnostic angiography if there was ongoing chest pain with or without dynamic ECG changes and/or further rise in troponin levels. |
| **Inclusion Criteria** | Patients ≥80 years old. Eligibility was predicated on the enrolling cardiologist confirming that the patient was suitable both for an intervention-guided or for an initially conservative strategy |
| **Exclusion Criteria** | Exclusion criteria included acute ST-elevation myocardial infarction (MI), cardiogenic shock, platelet count ≤50 × 109/mm3, life expectancy <1 year, allergies to antiplatelet therapy, major gastrointestinal haemorrhage within the preceding three months or any previous intracranial haemorrhage. |
| **Follow up visits** | Follow-up of well-being, drug compliance and events at 3 months, 6 months and 12 months. |
| **Participants** | 250 patients with NSTEMI aged ≥ 80 years |
| **Outcomes** | ***Primary***: composite of all-cause mortality and myocardial infarction at 12 months.  ***Secondary***: all-cause mortality, myocardial infarction, urgent revascularisation, stroke and bleeding at 12 months. |
| **Ethical Review** | The trial was approved by the National Research Ethics Service Committee South East Coast: Brighton and Sussex and the institutional review boards of each participating interventional centre. |
| **Overall bias assessment** | Low risk |

# Online Table 7 | Key Information: Sanchis et al., 2023 (MOSCA-FRAIL)

| **Principal Investigator** | **A. Ariza-Sole** |
| --- | --- |
| **Enrolment** | **Spain, July 2017 – January 2021** |
| **Study Design** | **Randomised controlled study**  ***‘Invasive’***. Coronary angiography within 72 hours of admission with coronary revascularization if deemed appropriate.  ***‘Conservative’*.** Medical therapy only (optimized according to the clinical practice guidelines recommendations), although cardiac catheterization was allowed in the case of recurrent ischemia during the index hospitalization. |
| **Inclusion Criteria** | The inclusion criteria were:  (1) NSTEMI;  (2) Age 70 years or older;  (3) Frailty criteria defined as scoring at least 4 points on the Rockwood Clinical Frailty Scale. |
| **Exclusion Criteria** | Exclusion criteria were prior known nonrevascularizable coronary artery disease, significant concomitant non-ischemic heart disease, inability to understand/sign informed consent (patients or relatives), and life expectancy less than 12 months. |
| **Follow up visits** | Clinic visit, electronic record review and telephone contact at 6 months and 1 year. |
| **Participants** | 167 patients with NSTEMI aged ≥ 70 years |
| **Outcomes** | ***Primary***: number of days alive and out of hospital at 12 months. Also a composite of cardiac death, reinfarction or revascularisation.  ***Secondary***: all-cause mortality, cardiac death, myocardial infarction, revascularisation and readmission due to cardiac causes or bleeding at 12 months. |
| **Ethical Review** | All centres received the approval of their Medical Ethics Committee |
| **Overall bias assessment** | Low risk |

# Online Table 8 | Demographics of participants across trials included in analysis

|  |  |  |  |  |  |  |
| --- | --- | --- | --- | --- | --- | --- |
| **Variable** | **After Eighty**  N = 457 | **Eighty+**  N = 186 | **MOSCA-FRAIL**  N = 167 | **Italian Elderly**  N = 313 | **MOSCA**  N = 106 | **RINCAL**  N = 250 |
| **Routine invasive treatment** | 229 (50%) | 93 (50%) | 84 (50%) | 154 (49%) | 52 (49%) | 124 (50%) |
| **Age** | 84.0 (82.0, 87.0) | 84.9 (82.6, 87.1) | 86.0 (82.0, 89.0) | 81.0 (77.0, 84.0) | 82.0 (78.0, 85.8) | 85.0 (82.0, 88.0) |
| **Male** | 236 (52%) | 102 (55%) | 79 (47%) | 157 (50%) | 56 (53%) | 132 (53%) |
| **Smoking status** |  |  |  |  |  |  |
| Smoker | 39 (8.5%) | 5 (2.7%) | 5 (3.0%) | 0 (0%) | 6 (5.7%) | 14 (5.6%) |
| Ex-smoker | 182 (40%) | 72 (39%) | 50 (30%) | 0 (0%) | 0 (0%) | 128 (51%) |
| Non-smoker | 215 (47%) | 100 (54%) | 112 (67%) | 0 (0%) | 100 (94%) | 103 (41%) |
| NA | 21 (4.6%) | 9 (4.8%) | 0 (0%) | 313 (100%) | 0 (0%) | 5 (2.0%) |
| **Angina** |  |  |  |  |  |  |
| No | 212 (46%) | 107 (58%) | 0 (0%) | 0 (0%) | 0 (0%) | 149 (60%) |
| Yes | 238 (52%) | 77 (41%) | 0 (0%) | 0 (0%) | 0 (0%) | 92 (37%) |
| NA | 7 (1.5%) | 2 (1.1%) | 167 (100%) | 313 (100%) | 106 (100%) | 9 (3.6%) |
| **Previous MI** |  |  |  |  |  |  |
| No | 256 (56%) | 120 (65%) | 116 (69%) | 216 (69%) | 59 (56%) | 178 (71%) |
| Yes | 195 (43%) | 64 (34%) | 51 (31%) | 97 (31%) | 47 (44%) | 68 (27%) |
| NA | 6 (1.3%) | 2 (1.1%) | 0 (0%) | 0 (0%) | 0 (0%) | 4 (1.6%) |
| **Previous CABG** |  |  |  |  |  |  |
| No | 376 (82%) | 153 (82%) | 151 (90%) | 284 (91%) | 92 (87%) | 225 (90%) |
| Yes | 75 (16%) | 33 (18%) | 16 (9.6%) | 29 (9.3%) | 14 (13%) | 22 (8.8%) |
| NA | 6 (1.3%) | 0 (0%) | 0 (0%) | 0 (0%) | 0 (0%) | 3 (1.2%) |
| **Previous PCI** |  |  |  |  |  |  |
| No | 350 (77%) | 154 (83%) | 115 (69%) | 266 (85%) | 85 (80%) | 209 (84%) |
| Yes | 101 (22%) | 31 (17%) | 52 (31%) | 47 (15%) | 21 (20%) | 37 (15%) |
| NA | 6 (1.3%) | 1 (0.5%) | 0 (0%) | 0 (0%) | 0 (0%) | 4 (1.6%) |
| **Hypertension** |  |  |  |  |  |  |
| No | 183 (40%) | 72 (39%) | 14 (8.4%) | 54 (17%) | 12 (11%) | 79 (32%) |
| Yes | 268 (59%) | 114 (61%) | 153 (92%) | 259 (83%) | 94 (89%) | 169 (68%) |
| NA | 6 (1.3%) | 0 (0%) | 0 (0%) | 0 (0%) | 0 (0%) | 2 (0.8%) |
| **Diabetes** |  |  |  |  |  |  |
| No | 372 (81%) | 150 (81%) | 74 (44%) | 199 (64%) | 57 (54%) | 197 (79%) |
| Yes | 79 (17%) | 36 (19%) | 93 (56%) | 114 (36%) | 49 (46%) | 52 (21%) |
| NA | 6 (1.3%) | 0 (0%) | 0 (0%) | 0 (0%) | 0 (0%) | 1 (0.4%) |
| **Previous stroke** |  |  |  |  |  |  |
| No | 383 (84%) | 161 (87%) | 137 (82%) | 288 (92%) | 80 (75%) | 197 (79%) |
| Yes | 68 (15%) | 25 (13%) | 30 (18%) | 25 (8.0%) | 26 (25%) | 51 (20%) |
| NA | 6 (1.3%) | 0 (0%) | 0 (0%) | 0 (0%) | 0 (0%) | 2 (0.8%) |
| **Peripheral arterial disease** |  |  |  |  |  |  |
| No | 403 (88%) | 177 (95%) | 149 (89%) | 0 (0%) | 61 (58%) | 242 (97%) |
| Yes | 48 (11%) | 9 (4.8%) | 18 (11%) | 0 (0%) | 45 (42%) | 5 (2.0%) |
| NA | 6 (1.3%) | 0 (0%) | 0 (0%) | 313 (100%) | 0 (0%) | 3 (1.2%) |
| **Atrial fibrillation** |  |  |  |  |  |  |
| No | 351 (77%) | 0 (0%) | 0 (0%) | 271 (87%) | 80 (75%) | 195 (78%) |
| Yes | 100 (22%) | 0 (0%) | 0 (0%) | 42 (13%) | 26 (25%) | 48 (19%) |
| NA | 6 (1.3%) | 186 (100%) | 167 (100%) | 0 (0%) | 0 (0%) | 7 (2.8%) |
| **Killip class** |  |  |  |  |  |  |
| 0 | 1 (0.2%) | 0 (0%) | 0 (0%) | 0 (0%) | 0 (0%) | 0 (0%) |
| 1 | 334 (73%) | 0 (0%) | 128 (77%) | 214 (68%) | 72 (68%) | 192 (77%) |
| 2 | 76 (17%) | 0 (0%) | 30 (18%) | 86 (27%) | 29 (27%) | 36 (14%) |
| 3 | 2 (0.4%) | 0 (0%) | 9 (5.4%) | 5 (1.6%) | 5 (4.7%) | 9 (3.6%) |
| 4 | 1 (0.2%) | 0 (0%) | 0 (0%) | 4 (1.3%) | 0 (0%) | 0 (0%) |
| NA | 43 (9.4%) | 186 (100%) | 0 (0%) | 4 (1.3%) | 0 (0%) | 13 (5.2%) |
| **Beta-blocker on discharge** |  |  |  |  |  |  |
| No | 222 (49%) | 20 (11%) | 38 (23%) | 128 (41%) | 36 (34%) | 104 (42%) |
| Yes | 229 (50%) | 163 (88%) | 121 (72%) | 185 (59%) | 63 (59%) | 146 (58%) |
| NA | 6 (1.3%) | 3 (1.6%) | 8 (4.8%) | 0 (0%) | 7 (6.6%) | 0 (0%) |
| **ACE inhibitor on discharge** |  |  |  |  |  |  |
| No | 310 (68%) | 98 (53%) | 61 (37%) | 119 (38%) | 20 (19%) | 146 (58%) |
| Yes | 138 (30%) | 84 (45%) | 98 (59%) | 194 (62%) | 79 (75%) | 104 (42%) |
| NA | 9 (2.0%) | 4 (2.2%) | 8 (4.8%) | 0 (0%) | 7 (6.6%) | 0 (0%) |
| **ARB on discharge** |  |  |  |  |  |  |
| No | 439 (96%) | 141 (76%) | 0 (0%) | 253 (81%) | 0 (0%) | 220 (88%) |
| Yes | 9 (2.0%) | 41 (22%) | 0 (0%) | 60 (19%) | 0 (0%) | 30 (12%) |
| NA | 9 (2.0%) | 4 (2.2%) | 167 (100%) | 0 (0%) | 106 (100%) | 0 (0%) |
| **Calcium channel blocker on discharge** |  |  |  |  |  |  |
| No | 341 (75%) | 144 (77%) | 0 (0%) | 223 (71%) | 0 (0%) | 229 (92%) |
| Yes | 107 (23%) | 37 (20%) | 0 (0%) | 90 (29%) | 0 (0%) | 21 (8.4%) |
| NA | 9 (2.0%) | 5 (2.7%) | 167 (100%) | 0 (0%) | 106 (100%) | 0 (0%) |
| **Short- or long-acting nitrate on discharge** |  |  |  |  |  |  |
| No | 262 (57%) | 31 (17%) | 0 (0%) | 132 (42%) | 0 (0%) | 144 (58%) |
| Yes | 186 (41%) | 148 (80%) | 0 (0%) | 181 (58%) | 0 (0%) | 106 (42%) |
| NA | 9 (2.0%) | 7 (3.8%) | 167 (100%) | 0 (0%) | 106 (100%) | 0 (0%) |
| **Aspirin on discharge** |  |  |  |  |  |  |
| No | 31 (6.8%) | 18 (9.7%) | 17 (10%) | 29 (9.3%) | 24 (23%) | 105 (42%) |
| Yes | 417 (91%) | 165 (89%) | 142 (85%) | 284 (91%) | 75 (71%) | 145 (58%) |
| NA | 9 (2.0%) | 3 (1.6%) | 8 (4.8%) | 0 (0%) | 7 (6.6%) | 0 (0%) |
| **Antiplatelet (P2Y12 inhibitor) on discharge** |  |  |  |  |  |  |
| No | 9 (2.0%) | 21 (11%) | 32 (19%) | 69 (22%) | 21 (20%) | 96 (38%) |
| Yes | 442 (97%) | 162 (87%) | 127 (76%) | 244 (78%) | 77 (73%) | 154 (62%) |
| NA | 6 (1.3%) | 3 (1.6%) | 8 (4.8%) | 0 (0%) | 8 (7.5%) | 0 (0%) |
| **Anticoagulant on discharge** |  |  |  |  |  |  |
| No | 376 (82%) | 154 (83%) | 113 (68%) | 297 (95%) | 0 (0%) | 214 (86%) |
| Yes | 81 (18%) | 24 (13%) | 46 (28%) | 16 (5.1%) | 0 (0%) | 36 (14%) |
| NA | 0 (0%) | 8 (4.3%) | 8 (4.8%) | 0 (0%) | 106 (100%) | 0 (0%) |
| **Statin on discharge** |  |  |  |  |  |  |
| No | 55 (12%) | 35 (19%) | 10 (6.0%) | 62 (20%) | 9 (8.5%) | 98 (39%) |
| Yes | 393 (86%) | 146 (78%) | 149 (89%) | 251 (80%) | 90 (85%) | 152 (61%) |
| NA | 9 (2.0%) | 5 (2.7%) | 8 (4.8%) | 0 (0%) | 7 (6.6%) | 0 (0%) |
| Median (IQR) or Frequency (%)  ACEi: angiotensin-converting-enzyme inhibitor. ARB: angiotensin II receptor blocker. CABG: coronary artery bypass graft. MI: myocardial infarction. NA: not available. P2Y12 inhibitor: purinergic receptor inhibitor antiplatelet. PCI: percutaneous coronary intervention. | | | | | | |

# Online Table 9 | Bleeding Events

| **Trial** | **Definition** | **Reported Findings** |
| --- | --- | --- |
| Savonitto et al., 2012  Italian Elderly ACS | BARC criteria ≥ 2 leading to hospitalisation, within 1 year | 2 (1%) severe bleeding events in invasive group and 1 (1%) severe bleeding events in conservative group. No analysis performed. |
| Tegn et al., 2016  After Eighty | Major or minor TIMI criteria, within median of 1.53 years | 4 (2%) major bleeding events in invasive group and 4 (2%) major bleeding events in conservative group. 23 (10%) minor bleeding events in invasive group and 16 (7%) minor bleeding events in conservative group. No analyses performed. |
| Sanchis et al., 2016  MOSCA | TIMI criteria ≥ 2, within median follow-up of 2.5 years | No significant difference between invasive (n=7, 13%) and conservative (n=10, 19%) groups. IRR 0.45; 95% CI 0.10-2.13, p=0.29. |
| Hirlekar et al., 2020  80+ Study | Major or minor TIMI criteria, within 1 month | No major bleeding events reported. No significant difference in minor bleeding events between invasive (n=4, 4%) and conservative (n=2, 2%) groups. HR 1.81; 95% CI 0.34-9.61, p=0.49. |
| De Belder et al., 2021  RINCAL | BARC criteria ≥ 3B, within 1 year | No significant difference in major bleeding events between invasive (n=7, 6%) and conservative groups (n=3, 2%); p=0.21. |
| Sanchis et al., 2023  MOSCA-FRAIL | Any bleeding requiring hospitalisation, within 1 year | Significantly higher rate of bleeding in invasive (n=8, 10%) compared to conservative (n=1, 1%) group. IRR 14.9; 95% CI 1.7-12.9, p=0.02. |
| BARC, Bleeding Academic Research Consortium. CI, confidence interval. HR, hazard ratio. IRR, incidence rate ratio. TIMI, Thrombolysis In Myocardial Infarction. | | |

# Online Table 10 | Use of Radial Access and Drug-Eluting Stents

| **Trial** | **Radial Access (%)*** | **Drug-Eluting Stents (%)^†^** |
| --- | --- | --- |
| Savonitto et al., 2012  Italian Elderly ACS | 73 | 53 |
| Tegn et al., 2016  After Eighty | 90 | 37 |
| Sanchis et al., 2016  MOSCA | 91 | 47 |
| Hirlekar et al., 2020  80+ Study | *‘High proportion of radial access’* | 44 |
| De Belder et al., 2021  RINCAL | 82 | 82 |
| Sanchis et al., 2023  MOSCA-FRAIL | 86 | 90 |
| * as percentage of coronary angiograms  **^†^** as percentage of percutaneous coronary interventions. Quantitative data on the use of radial access in the 80+ Study was not available. | | |

# Online Figure 1 | Risk of bias assessment

Qualitative assessment of the risk of bias of the included randomised controlled trials using the Cochrane Collaborative method. Green indicates low risk of bias, red indicates risk of bias and yellow indicates unclear risk. Blinding of participants and personnel to the intervention was not possible given the nature of the intervention – this was common across all trials but remains a limitation.

# Online Figure 2 | Adverse outcomes one year post randomisation.

Plots presenting HRs and 95% CI intervals for the comparison of participants in the invasive group (IG) versus the conservative group (CG) using fixed effect Cox models to adjust for within-study clustering. The numbers in the IG and CG columns represent the number of events/total number of participants in each group. Denominators are different for CV death, due to missingness of this outcome in one study, and for the revascularisation endpoint, because 6 participants were missing follow-up time values. The composite endpoint includes all-cause mortality and/or myocardial infarction. IG: invasive group; CG: conservative group; CV death: cardiovascular death; HR: hazard ratio; CI: confidence interval.

# Online Figure 3 | Random effects forest plots for individual studies

Plots presenting HRs and 95% CI intervals for the individual trials comparing the invasive group versus the conservative groups using two stage meta-analysis. Heterogeneity is presented by the I^2^ value.

# Online Figure 4 | Sensitivity analyses for adverse outcomes one year post randomisation

Plots presenting adjusted HRs and 95% CI intervals for the comparison of participants in the invasive group (IG) versus the conservative group (CG) using random effects (**black font**) and fixed effect (**blue font**) Cox models to adjust for within-study clustering, further adjusted for age, sex, hypertension, and diabetes. The numbers in the IG and CG columns represent the number of events/total number of participants in each group. Denominators are different for CV death, due to missingness of this outcome in one study, and for the revascularisation endpoint, because 6 participants were missing follow-up time values. The composite endpoint includes all-cause mortality and/or myocardial infarction. IG: invasive group; CG: conservative group; CV death: cardiovascular death; HR: hazard ratio; CI: confidence interval.

# Online Figure 5 | Sensitivity analysis excluding peri-procedural MI

Total number of myocardial infarction events and peri-procedural myocardial infarction (PMI) per study per randomisation group. PMI was defined according to the Type 4a universal definition of MI. For the conservative group, patients that crossovered to undergo coronary angiography were only considered. All PMI was defined as occurring within 2 days (48 hours) post randomisation (A). Plots presenting HRs and 95% CI intervals for the comparison of participants in the invasive group (IG) versus the conservative group (CG) using random effects Cox models to adjust for within-study clustering for the MI and MI excluding PMI endpoints.

# Online Figure 6 | Meta-regression analysis of crossover rates

Scatterplot by the rate of crossover to invasive treatment (both coronary angiography and subsequent revascularisation) of patients in the conservative group, using the hazard ratio for the composite endpoint of all-cause mortality and repeat myocardial infarction. The size of each study point is a function of the square root of the relative model weight. No rate was available for the After Eighty trial.

# Online Figure 7 | Adverse outcomes one year post randomisation excluding crossover patients.

Plots presenting HRs and 95% CI intervals for the comparison of participants in the invasive group (IG) versus the conservative group (CG) **excluding those that underwent coronary angiography** using **random** effects Cox models to adjust for within-study clustering. The numbers in the IG and CG columns represent the number of events/total number of participants in each group. The composite endpoint includes all-cause mortality and/or myocardial infarction. IG: invasive group; CG: conservative group; CV death: cardiovascular death; HR: hazard ratio; CI: confidence interval.

# Online Figure 8 | Meta-regression analysis of publication year

Scatterplot by publication year using the hazard ratio for the composite endpoint of all-cause mortality and repeat myocardial infarction. The size of each study point is a function of the square root of the relative model weight.

# Online Figure 9 | Funnel plot.

Funnel plot to visualise any potential publication bias for the composite endpoint.

# PRISMA-IPD Checklist

**PRISMA-IPD Checklist of items to include when reporting a systematic review and meta-analysis of individual participant data (IPD)**

| **PRISMA-IPD**  **Section/topic** | **Item No** | **Checklist item** | **Reported on page** |
| --- | --- | --- | --- |
| **Title** | | | |
| Title | 1 | Identify the report as a systematic review and meta-analysis of individual participant data. | P1 |
| **Abstract** | | | |
| Structured summary | 2 | Provide a structured summary including as applicable: | P2/3 |
|  |  | **Background**: state research question and main objectives, with information on participants, interventions, comparators and outcomes. |  |
|  |  | **Methods**: report eligibility criteria; data sources including dates of last bibliographic search or elicitation, noting that IPD were sought; methods of assessing risk of bias. |  |
|  |  | **Results**: provide number and type of studies and participants identified and number (%) obtained; summary effect estimates for main outcomes (benefits and harms) with confidence intervals and measures of statistical heterogeneity. Describe the direction and size of summary effects in terms meaningful to those who would put findings into practice. |  |
|  |  | **Discussion:** state main strengths and limitations of the evidence, general interpretation of the results and any important implications. |  |
|  |  | **Other:** report primary funding source, registration number and registry name for the systematic review and IPD meta-analysis. |  |
| **Introduction** | | | |
| Rationale | 3 | Describe the rationale for the review in the context of what is already known. | P5 |
| Objectives | 4 | Provide an explicit statement of the questions being addressed with reference, as applicable, to participants, interventions, comparisons, outcomes and study design (PICOS). Include any hypotheses that relate to particular types of participant-level subgroups. | P6 |
| **Methods** | | | |
| Protocol and registration | 5 | Indicate if a protocol exists and where it can be accessed. If available, provide registration information including registration number and registry name. Provide publication details, if applicable. | P7 |
| Eligibility criteria | 6 | Specify inclusion and exclusion criteria including those relating to participants, interventions, comparisons, outcomes, study design and characteristics (e.g. years when conducted, required minimum follow-up). Note whether these were applied at the study or individual level i.e. whether eligible participants were included (and ineligible participants excluded) from a study that included a wider population than specified by the review inclusion criteria. The rationale for criteria should be stated. | P7 |
| Identifying studies - information sources | 7 | Describe all methods of identifying published and unpublished studies including, as applicable: which bibliographic databases were searched with dates of coverage; details of any hand searching including of conference proceedings; use of study registers and agency or company databases; contact with the original research team and experts in the field; open adverts and surveys. Give the date of last search or elicitation. | P7 |
| Identifying studies - search | 8 | Present the full electronic search strategy for at least one database, including any limits used, such that it could be repeated. | Appendix p3 |
| Study selection processes | 9 | State the process for determining which studies were eligible for inclusion. | P7 |
| Data collection processes | 10 | Describe how IPD were requested, collected and managed, including any processes for querying and confirming data with investigators. If IPD were not sought from any eligible study, the reason for this should be stated (for each such study). | P7 |
|  |  | If applicable, describe how any studies for which IPD were not available were dealt with. This should include whether, how and what aggregate data were sought or extracted from study reports and publications (such as extracting data independently in duplicate) and any processes for obtaining and confirming these data with investigators. |  |
| Data items | 11 | Describe how the information and variables to be collected were chosen. List and define all study level and participant level data that were sought, including baseline and follow-up information. If applicable, describe methods of standardising or translating variables within the IPD datasets to ensure common scales or measurements across studies. | P8 |
| IPD integrity | A1 | Describe what aspects of IPD were subject to data checking (such as sequence generation, data consistency and completeness, baseline imbalance) and how this was done. | P8 |
| Risk of bias assessment in individual studies. | 12 | Describe methods used to assess risk of bias in the individual studies and whether this was applied separately for each outcome. If applicable, describe how findings of IPD checking were used to inform the assessment. Report if and how risk of bias assessment was used in any data synthesis. | P9 |
| Specification of outcomes and effect measures | 13 | State all treatment comparisons of interests. State all outcomes addressed and define them in detail. State whether they were pre-specified for the review and, if applicable, whether they were primary/main or secondary/additional outcomes. Give the principal measures of effect (such as risk ratio, hazard ratio, difference in means) used for each outcome. | P8/9 |
| Synthesis methods | 14 | Describe the meta-analysis methods used to synthesise IPD. Specify any statistical methods and models used. Issues should include (but are not restricted to):   - Use of a one-stage or two-stage approach. - How effect estimates were generated separately within each study and combined across studies (where applicable). - Specification of one-stage models (where applicable) including how clustering of patients within studies was accounted for. - Use of fixed or random effects models and any other model assumptions, such as proportional hazards. - How (summary) survival curves were generated (where applicable). - Methods for quantifying statistical heterogeneity (such as I^2^ and τ^2^). - How studies providing IPD and not providing IPD were analysed together (where applicable). - How missing data within the IPD were dealt with (where applicable). | P9/10 |
| Exploration of variation in effects | A2 | If applicable, describe any methods used to explore variation in effects by study or participant level characteristics (such as estimation of interactions between effect and covariates). State all participant-level characteristics that were analysed as potential effect modifiers, and whether these were pre-specified. | P9/10 |
| Risk of bias across studies | 15 | Specify any assessment of risk of bias relating to the accumulated body of evidence, including any pertaining to not obtaining IPD for particular studies, outcomes or other variables. | - |
| Additional analyses | 16 | Describe methods of any additional analyses, including sensitivity analyses. State which of these were pre-specified. | P9/10 |
| **Results** | | | |
| Study selection and IPD obtained | 17 | Give numbers of studies screened, assessed for eligibility, and included in the systematic review with reasons for exclusions at each stage. Indicate the number of studies and participants for which IPD were sought and for which IPD were obtained. For those studies where IPD were not available, give the numbers of studies and participants for which aggregate data were available. Report reasons for non-availability of IPD. Include a flow diagram. | P11 |
| Study characteristics | 18 | For each study, present information on key study and participant characteristics (such as description of interventions, numbers of participants, demographic data, unavailability of outcomes, funding source, and if applicable duration of follow-up). Provide (main) citations for each study. Where applicable, also report similar study characteristics for any studies not providing IPD. | P11 & Appendix P7-18 |
| IPD integrity | A3 | Report any important issues identified in checking IPD or state that there were none. | Appendix P3 |
| Risk of bias within studies | 19 | Present data on risk of bias assessments. If applicable, describe whether data checking led to the up-weighting or down-weighting of these assessments. Consider how any potential bias impacts on the robustness of meta-analysis conclusions. | P11 & Appendix P21 |
| Results of individual studies | 20 | For each comparison and for each main outcome (benefit or harm), for each individual study report the number of eligible participants for which data were obtained and show simple summary data for each intervention group (including, where applicable, the number of events), effect estimates and confidence intervals. These may be tabulated or included on a forest plot. | P11 & Figures |
| Results of syntheses | 21 | Present summary effects for each meta-analysis undertaken, including confidence intervals and measures of statistical heterogeneity. State whether the analysis was pre-specified, and report the numbers of studies and participants and, where applicable, the number of events on which it is based. | P11/12 & Figures |
|  |  | When exploring variation in effects due to patient or study characteristics, present summary interaction estimates for each characteristic examined, including confidence intervals and measures of statistical heterogeneity. State whether the analysis was pre-specified. State whether any interaction is consistent across trials. |  |
|  |  | Provide a description of the direction and size of effect in terms meaningful to those who would put findings into practice. |  |
| Risk of bias across studies | 22 | Present results of any assessment of risk of bias relating to the accumulated body of evidence, including any pertaining to the availability and representativeness of available studies, outcomes or other variables. | - |
| Additional analyses | 23 | Give results of any additional analyses (e.g. sensitivity analyses). If applicable, this should also include any analyses that incorporate aggregate data for studies that do not have IPD. If applicable, summarise the main meta-analysis results following the inclusion or exclusion of studies for which IPD were not available. | P13/14 |
| **Discussion** | | | |
| Summary of evidence | 24 | Summarise the main findings, including the strength of evidence for each main outcome. | P15-18 |
| Strengths and limitations | 25 | Discuss any important strengths and limitations of the evidence including the benefits of access to IPD and any limitations arising from IPD that were not available. | P19-20 |
| Conclusions | 26 | Provide a general interpretation of the findings in the context of other evidence. | P20 |
| Implications | A4 | Consider relevance to key groups (such as policy makers, service providers and service users). Consider implications for future research. | P20 |
| **Funding** | | | |
| Funding | 27 | Describe sources of funding and other support (such as supply of IPD), and the role in the systematic review of those providing such support. | P10/22/ 24 |

**A1 – A3 denote new items that are additional to standard PRISMA items. A4 has been created as a result of re-arranging content of the standard PRISMA statement to suit the way that systematic review IPD meta-analyses are reported.**

© Reproduced with permission of the PRISMA IPD Group, which encourages sharing and reuse for non-commercial purposes
